# A Case for Antibodies as Mechanistic Correlates of Immunity in Tuberculosis

**DOI:** 10.3389/fimmu.2019.00996

**Published:** 2019-05-09

**Authors:** Jeffrey Y. Kawahara, Edward B. Irvine, Galit Alter

**Affiliations:** ^1^Ragon Institute of MGH, MIT and Harvard, Massachusetts Institute of Technology, Cambridge, MA, United States; ^2^Harvard Medical School, Boston, MA, United States; ^3^Harvard T.H. Chan School of Public Health, Boston, MA, United States

**Keywords:** tuberculosis, antibodies, cell-mediated immunity, Fc effector function, innate immune system, humoral immunity

## Abstract

Tuberculosis infects one quarter of the world's population and is the leading cause of death by a single infectious agent, responsible for a reported 1.3 million deaths in 2017. While *Mycobacterium tuberculosis* is treatable with antibiotic therapy, the increased prevalence of drug resistance, coupled with the variable efficacy of the only widely approved vaccine, has highlighted the need for creative approaches to therapeutic and vaccine development. Historically, a productive immune response to *M. tuberculosis* has been thought to be nearly entirely cell-mediated, with humoral immunity being largely dismissed. However, in this review, we will discuss the historical skepticism surrounding the role of the humoral immune response to *M. tuberculosis*, and examine more recent evidence suggesting that antibodies may play a valuable role in host defense against the pathogen. Despite the amount of data portraying antibodies in a negative light, emerging data have begun to highlight the unexpected role of antibodies in *M. tuberculosis* control. Specifically, it has become clear that antibody features of both the variable and constant domain (Fc) ultimately determine the extent to which antibodies modulate disease. Thus, a more precise definition of the antigen-binding and innate immune recruiting functions of antibodies that contribute to *M. tuberculosis* restriction, are sure to help guide the development of next-generation therapeutics and vaccines to curb this global epidemic.

## Introduction

*Mycobacterium tuberculosis* (*Mtb*), the causative agent of tuberculosis, is the leading cause of death from single infectious agent globally. *Mtb* infects one quarter of the global population, and caused ~1.3 million deaths worldwide in 2017 (1). Importantly, tuberculosis infection does not always lead to tuberculosis disease, as tuberculosis presents as a spectrum of infection states. These range from an asymptomatic state, referred to as latent infection (LTBI), to the deadlier active disease (ATB).

While tuberculosis is treatable with antibiotics, the immense global burden, as well as the rise of drug resistance, has highlighted the need for improved methods for disease treatment and prevention. Bacillus Calmette-Guérin (BCG), developed nearly a century ago, remains the only licensed tuberculosis vaccine. Prepared using a live attenuated strain of *Mycobacterium bovis*, its protective efficacy is remarkably inconsistent (2, 3). BCG shows consistent protection against severe forms of tuberculosis disease, such as tuberculosis meningitis and miliary tuberculosis, in infants (4). However, the vaccine exhibits limited protection against pulmonary tuberculosis, and importantly, does not protect teenagers and adults who are most likely to spread *Mtb* (5, 6). Given that BCG vaccination is widely given, yet tuberculosis remains the largest infectious disease killer globally, it is clear that a more effective vaccine is urgently needed to control the disease globally. Thus, creative approaches to therapeutic and vaccine development are critical to change the trajectory of the ongoing tuberculosis epidemic.

Cellular-mediated immunity (CMI), in particular CD4^+^ T cells, are unequivocally important in restricting tuberculosis progression, and are seen as the primary immunologic axis mediating host immunity to *Mtb*. Both CD4 knock-out studies in animal models (7–9), as well as epidemiologic data documenting increased rates of active disease among HIV-infected patients with low CD4^+^ T cell counts (10, 11), clearly demonstrate the lack of bacterial control in the absence of this pivotal immune effector. Thus, historically, the vast majority of vaccine design efforts have focused on the development of strategies that harness T cell immunity to drive protection or control of *Mtb*.

Conversely, while antibodies represent the correlate of immunity following most clinically approved vaccines (12), humoral immunity has been understudied in the context of *Mtb* vaccine design due to its perceived insignificance for anti-microbial control (13–15). Yet, the idea that the humoral immune response plays little role in *Mtb* infection is in part related to the perceived dichotomy between humoral and cellular immunity. Specifically, the paradigm dictates that Th1 responses counter intracellular pathogens by driving CMI, while humoral immunity is largely responsible for the control and clearance of extracellular pathogens (16). Consequently, in the absence of unambiguous evidence proving a protective role for antibodies, it has been assumed that due to their extracellular canonical mode of action, antibodies must not be relevant or critical for protection against *Mtb*. Moreover, despite our emerging appreciation for a role for antibodies in driving cellular cytotoxicity via the recruitment of the innate immune system as well as additional anti-microbial mechanisms, the perceived insignificance of the humoral immune response to *Mtb* remains pervasive in the field.

However, a growing body of literature has provided evidence indicating that *Mtb*-specific antibodies modulate tuberculosis disease. Specifically, evidence from passive transfer, monoclonal therapeutic, cohort, and vaccine studies each individually, and collectively argue that antibodies can positively shape the immune response to *Mtb*. Here we will discuss the uncertainties that have long surrounded the antibody response to *Mtb*, as well as examine the evidence suggesting that antibodies represent a wealth of untapped potential against this global killer.

## A Case for Antibodies From Passive Transfer Studies

The positive results of serum therapies against a range of infectious diseases in the late 1800s spawned a plethora of human and animal transfer experiments attempting to cure tuberculosis by the same methodology. In the 1890s, the Henry Phipps Institute immunized cows with a heat-killed concentrate of *Mtb* bacilli (17). However, the administration of the cow serum failed to show any benefit in tuberculosis patients (18, 19). Similar work performed by Viquerat and De Schweinitz aimed at exploring the impact of administration of immune sera from different animals (horse, cow, donkey) on disease, again, showed little benefit following passive transfer (18, 19). Moreover, in a more recent study, New Zealand rabbits were intravenously infected with *Mtb* in order to generate immune serum. When this serum was administered to mice challenged with BCG, disease was actually enhanced (20), arguing for a deleterious effect of *Mtb*-specific antibodies. Over time, studies, such as these have helped to construct the narrative that antibodies are not beneficial, and may even be detrimental.

Yet, in the wake of these disappointments, several studies had in fact shown a beneficial, bactericidal activity of antibodies both *in vitro* and *in vivo*. For example, immune guinea pig serum was reported to have complete bactericidal activity against *Mtb in vitro* (18). Moreover, early passive transfer of immune bovine serum in 412 subjects with tuberculosis, was reported to induce complete resolution of disease in 16% of treated subjects, to ameliorate clinical symptoms in 40% of subjects, and to mediate the clearance of sputum bacteria in 43% of patients treated with serum (21). Similarly, immune donkey serum was also reported to cure 83% of treated subjects in another study (22). Finally, the use of horse serum was shown to have significant disease benefit in more than 80% of treated individuals in one study, however the same serum had limited benefit in additional clinical studies (23, 24), calling into question comparability across studies (19). Thus, in reality, small sample sizes, differences in disease severity, differences in clinical endpoint analyses, and the lack of control groups in many of these passive transfer studies resulted in mixed findings, ranging from no benefit to complete resolution of disease (6, 19, 22–24). Furthermore, antibiotics began to gain traction at this time showing consistent anti-microbial effects (25, 26), casting further doubt, not on the anti-microbial activity of some sera, but in the utility of these therapeutics in light of simpler treatment regimens with drugs.

Yet, despite the intermittent signals of efficacy, little attention focused on the underlying biological differences across studies. Specifically, little attention was paid to potential species-specific differences in efficacy across passively transferred antibodies, the impact of sensitization approaches, the critical potential impact of differences in antibody-specificities across immune sera, or the overall anti-microbial potency of the transferred sera. Thus, rather than demonstrating a lack of clinical benefit, collectively, the body of passive transfer studies instead clearly highlighted that not all antibodies are protective, and that qualitative nuances exist across humoral responses that are critical determinants of humoral protection (19).

Despite the confusing historical data, a number of passive transfer studies performed in the past two decades support the protective nature of serum-transfer. In a passive transfer study by Guirado et al., a liposome packaged preparation of *Mtb* extract in combination with rifampicin and isoniazid was administered to *Mtb* infected mice (27). Hyperimmune serum was then extracted from these mice and applied to previously *Mtb* infected SCID mice (lacking functional T cell, B cells, and NK cells) that had received antibiotics. Through 10 weeks post-infection, the hyperimmune serum treated mice maintained lower CFUs in the lungs than control groups (27). Thus, even in the setting of a compromised immune response in the SCID mice, passive transfer was able to reduce *Mtb* burden *in vivo* independent of CMI.

Another significant passive transfer phenotype resulted from the administration of human intravenous immunoglobulin (IVIg) to *Mtb*-infected mice. IVIg is derived from the serum of large numbers of individuals, and often harbors immunoglobulin from *Mtb* exposed individuals either due to BCG vaccination, undiagnosed latent infection, or environmental mycobacterial exposure. Using an IVIg preparation with *Mtb*-specific antibodies, investigators observed a significant decrease in *Mtb* burden in mice following the passive administration of IVIg (28). Interestingly, this anti-microbial function was observed when the pool of antibodies was administered both early and late in infection (28). Given the emerging appreciation for the critical protective role of antibody IVIg Fc-glycosylation in autoimmune disease (29), Olivares et al. profiled the impact of Fc-deglycosylated IVIg. Removal of the Fc-glycan using EndoS, resulted in significantly reduced anti-microbial control, highlighting for the first time that the Fc-activity of transferred antibodies was critical to their protective function (30). Prior work by Olivares et al. found that intranasal IVIg was protective in mice and that this effect was eliminated when the inoculant was depleted of *Mtb*-specific antibodies, indicating that protection was mediated by antibody binding and subsequent Fc-receptor interactions (31). Thus, these data pointed to functions beyond simple binding and blockade of infection, in control and elimination of *Mtb*.

Finally, an intriguing study by Li et al. demonstrated that some, but not all, sera from infected or even sensitized-but-uninfected individuals harbor protective antibodies in serum transfer studies (32). Specifically, sera from healthcare workers with latent infection or from healthcare workers that were highly exposed, but remained negative in clinical diagnostic tests, were transferred to mice prior to aerosol challenge. Upon challenge, antibodies from half a dozen healthcare workers, including both latently and uninfected donors, conferred protection, resulting in a 2- to 3-fold decrease in lung CFUs compared to mice receiving serum from actively infected donors. Surprisingly, CD4^+^ T cells were also critical for serum-based protection, suggesting a novel axis by which antibodies and T cells may work synergistically to drive anti-microbial function. Moreover, beyond this observation, the data reported from this study were the first to highlight that not all sera are equally protective, and that even sera from highly-exposed, but uninfected individuals may harbor the key protective humoral immune responses that can drive anti-microbial function *in vivo*.

Taken together, despite early inconsistencies in preclinical and clinical results following polyclonal passive transfer studies, emerging data clearly highlight the protective nature of some—but not all—serum preparations. These data clearly motivate a re-examination of the specific features of polyclonal antibody responses able to drive protection following serum transfer.

## A Case for Antibodies From Monoclonal Therapeutic Studies

Due to their defined antigen specificity and modifiable Fc domains, monoclonal antibodies allow the precise determination of antibody features contributing to disease control. In the late 1990's, an array of murine-derived monoclonal antibodies were created and assessed for their ability to mediate protection in mice that largely exhibited progressive disease (33), representing a high bar for antibody-mediated protection. While not all tested monoclonal antibodies conferred protection *in vivo*, antibody clone 9d8, specific to the capsular antigen arabinomannan (AM), significantly improved survival over time (34). While this antibody did not reduce *Mtb* burden, it prolonged survival via improved granulomatous containment of *Mtb*. Interestingly, rather than a canonical blocking function of the antibody, the authors speculated that this effect was attributable to antibody-mediated enhancement of cellular immunity. These data were among the first to highlight the protective activity of monoclonal antibodies, as well as the possibility that these molecules may confer protection through unexpected mechanisms of action.

Since this study, additional monoclonal antibody passive transfer experiments with antibodies targeting different *Mtb* antigens have resulted in various forms of protective activity. Hamasur et al. observed that antibody clone SMITB14, specific to the cell well-glycolipid lipoarabinomannan (LAM), prolonged survival upon administration to mice (35). In contrast to the previous study with the 9d8 antibody to AM, the LAM-specific SMITB14 antibody resulted in a significant reduction in bacterial burden in the lungs and spleen of infected mice. Importantly, when the F(ab′)_2_ domain of SMITB14 was administered, following the removal of the antibody constant domain that is required for the recruitment of innate immune functions, survival was similar to full length antibodies, suggesting that the binding activity of the antibody was sufficient to provide protection against disease. In contrast to the work with IVIg, this data pointed to the importance of binding and potential blockade of infection by LAM-specific antibodies, analogous to the neutralization of viruses (36), that is independent of Fc-interactions within the immune system (35).

Two antibodies targeting *Mtb* protein antigens have additionally been shown to modulate disease *in vivo*. Passive transfer of a monoclonal antibody binding heparin-binding hemagglutinin adhesin (HBHA), a surface exposed mycobacterial adhesin (37, 38), was shown to prevent mycobacterial extrapulmonary dissemination in mice (38). Additionally, a second antibody targeting the heat shock protein X (HspX), a stress-induced intracellular and cell wall protein (39), was shown to reduce lung *Mtb* burden in mice (40). Interestingly, the HspX-specific antibody was of the human IgA1 isotype, and importantly, protection was only observed in mice transgenic for human CD89 (FcαR1), the primary receptor for human IgA. Monoclonal antibody administered to the CD89-negative littermates did not demonstrate any measurable level of protection, strongly suggesting that the effect was Fc-mediated.

**Figure 1:**
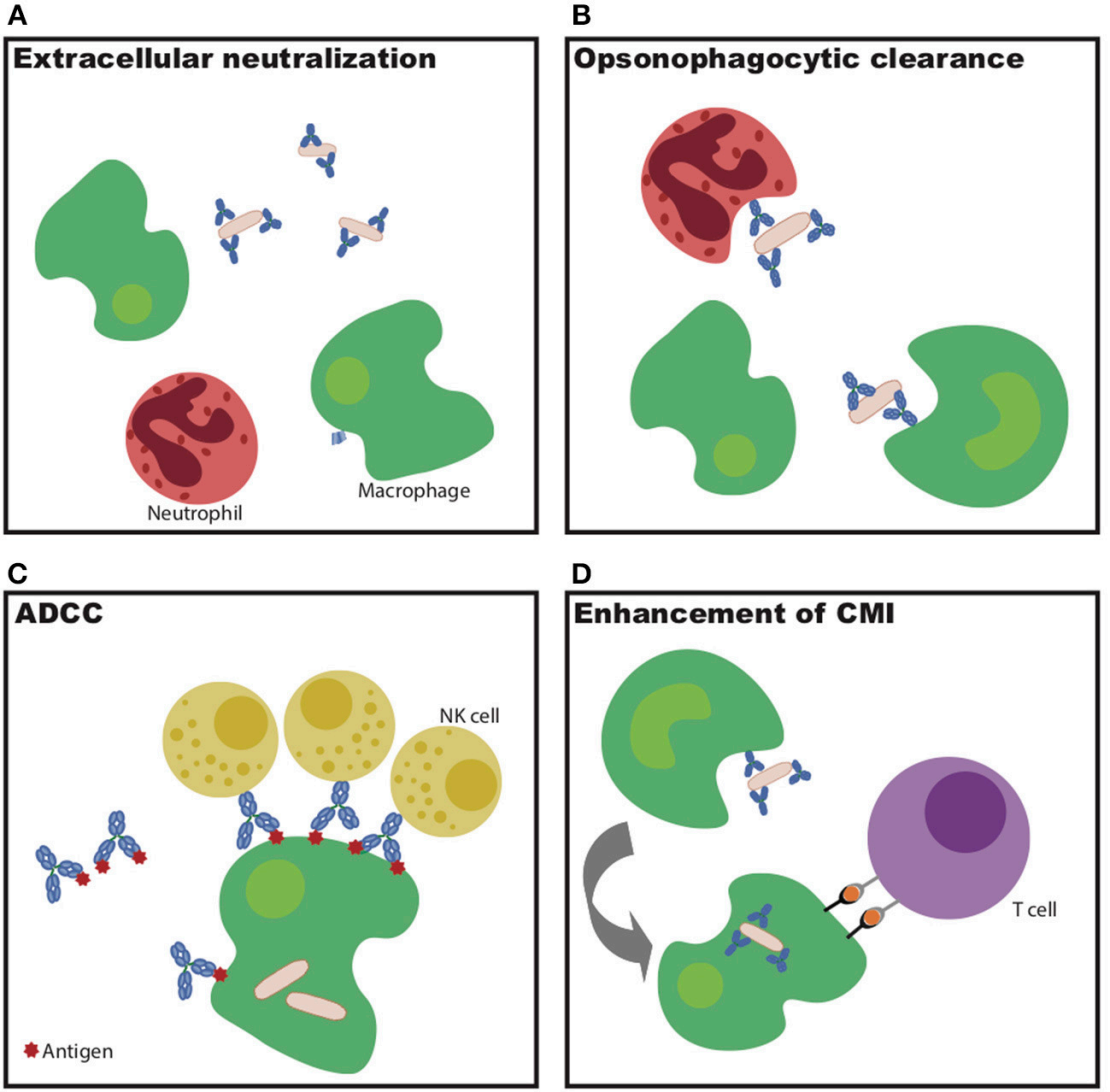
Potential mechanisms of antibody-mediated Mtb restriction. **(A)** Antibody binding to extracellular bacteria prevents entry into cells. **(B)** Antibody-dependent cellular phagocytosis drives increased bacterial killing during uptake. **(C)** Antibody-dependent cellular cytotoxicity drives infected cell and/or bacterial killing. **(D)** Antibodies potentiate cell-mediated immunity via enhanced antigen presentation.

Overall, this monoclonal therapeutic work demonstrates that multiple antibody specificities are able to confer protection against *Mtb in vivo*. However, the protective mechanisms diverge by specificity, highlighting the multiple humoral mechanisms—some via strict blockade and others via innate immune engagement (Figure 1)—that may be harnessed to control and ultimately eliminate the bacteria *in vivo*.

## A Case for Antibodies From Cohort Studies

Arguments for and against antibodies have additionally emerged based on observations from human cohort studies. A study in China showed that patients with X-linked agammaglobulinemia (XLA) do not have increased susceptibility to tuberculosis despite lacking mature B cells and normal antibody titers (41). Similarly, patients with defective Bruton's tyrosine kinase genes, manifesting in compromised humoral immunity, also show no indication that humoral immune deficiencies predispose individuals to increased risk of tuberculosis disease (12, 42). However, critically, many of the study subjects, lacking humoral immune components, were given IVIg therapy (12, 41, 42), transferring *Mtb*-specific antibodies; a clear confounding factor given the protective effect of IVIg described above (28, 30, 31). Other arguments against humoral immunity cite the finding that humans receiving B cell-depleting monoclonal antibody, rituximab, do not have a measurable increase in risk for tuberculosis disease (43, 44). While appealing, the argument does not take into consideration the fact that rituximab has a limited to negligible impact on depleting antibody-secreting cells, plasma cells, that reside in the bone marrow and do not express CD20. Moreover, rituximab treatment does not alter antibody levels that remain relatively stable over time (43, 44).

In contrast to the arguments against antibodies as correlates of *Mtb* control, both historical and more recent cohort studies have indicated a potential role for antibodies in combating tuberculosis disease. While increased *Mtb*-specific antibody levels have traditionally been associated with progressive disease (12), in the 1990s, a study in children from the UK and southeast Asia found that lipoarabinomannan (LAM)-specific IgG titers correlated with decreased risk of disseminated disease independent of age and geographic origin (45). Troughs in LAM IgG titer levels coincided with peak incidence of bacterial dissemination, and serum derived from the disseminated group also showed significantly (*p* < 0.05) decreased titers to purified LAM as measured by LAM binding ELISA. These data pointed to specific antibody populations, rather than total *Mtb*-specific antibodies, as a correlate of disease control.

More recently, building on qualitative differences in *Mtb*-specific antibodies, it was observed that individuals with latent and active tuberculosis generate distinct functional antibody profiles (46). Given the ability of antibodies to deploy the innate immune system to drive pathogen clearance, the study broadly profiled the Fc-profile of *Mtb*-specific antibodies. The study noted nearly distinct antibody Fc profiles across the groups, with differences largely driven by distinct IgG Fc-glycosylation patterns. Specifically, purified IgG from LTBI patients exhibited enhanced binding levels to FcγRIIIa, the Fc-receptor found on NK cells, which resulted in increased antibody-dependent cellular cytotoxicity (ADCC) and increased NK cell activation. Intriguingly, these differences in biophysical and functional differences also corresponded with an increased ability for antibodies from LTBI patients to drive intracellular *Mtb* killing in macrophages. Consistent with these data, a recent multi-cohort analysis that aimed to identify immune factors associated with asymptomatic LTBI, observed that LTBI control was associated with higher signaling via FcγRIIIa, and enhanced NK cell activity (47), corroborating the potential role of NK cell recruiting antibodies in long-term *Mtb* control.

Taken together, while human cohort studies have been commonly pointed to as evidence against a protective role for antibodies in *Mtb* infection, increasing evidence argues against this. Human cohort studies have been consistent with monoclonal and passive transfer studies, identifying LAM as a productive antibody target, as well as identifying the Fc-domain and antibody effector functions as critical qualities that modulate disease outcome. Thus, specifically manipulating both the specificity and functionality of the *Mtb*-specific humoral immune response represent tractable approaches for the design of next-generation therapeutics and vaccines.

## A Case for Antibodies From Vaccine Studies

Despite the fact that nearly all successful vaccines to date function by eliciting protective antibody responses, the majority of tuberculosis vaccines in use and in development, have been focused on the induction of CMI (48). Recent work by Hansen et al. demonstrates that a cytomegalovirus vector vaccine expressing *Mtb* antigens protects against *Mtb* infection without eliciting detectable antibody levels in the blood (49). However, a number of vaccine studies in murine and macaque preclinical models, as well as large-scale clinical trials in humans, have suggested a potentially protective role for antibodies in *Mtb* vaccine efficacy.

Beginning in the murine model, Prados-Rosales et al. performed one of the few studies that rationally designed a tuberculosis vaccine to selectively induce a mycobacteria-specific antibody response (50). Specifically, the capsular polysaccharide AM was conjugated to either *Mtb* Ag85b or the *B. anthracis* protective antigen. Both polysaccharide-conjugate vaccines significantly reduced lung bacterial burden in *Mtb* infected mice. Additionally, passive transfer of immune serum from AM-vaccinated mice provided protection in the form of reduced lung bacterial burden when administered prior to *Mtb* challenge of naïve mice. These data therefore provided concrete evidence for a protective role of vaccine induced AM-specific antibodies *in vivo*, pointing to the potential utility of a polysaccharide conjugate vaccine against *Mtb*.

In the rhesus macaque model, a localized, lung specific vaccine-induced antibody response was linked to protection (51). Specifically, rhesus macaques were vaccinated with BCG either intradermally, representing the standard route of immunization, or by the mucosal route via endobronchial instillation. Following repeated low-dose challenge with *Mtb*, the group receiving the mucosal vaccination demonstrated decreased lung CFUs and pathology. Intriguingly, the mucosal group also mounted a unique and robust PPD-specific antibody response, including enhanced PPD-specific IgA levels, locally in the bronchoalveolar lavage fluid. This compartment-specific induction of immunoglobulin in the lung represented a primary correlate of protective immunity, pointing to a humoral correlate of protection—focused at the site of infection—for the first time following BCG vaccination.

Finally, in humans, antibodies have emerged as a correlate of protection in a number of different tuberculosis vaccine clinical trials. In 2009, a phase 2b trial was conducted in BCG primed individuals, using a recombinant Vaccinia Ankara virus modified to express Ag85A (MVA85A) to boost cellular immune responses (52). While results confirmed that the treatment was safe and tolerable, vaccine efficacy was not significantly higher than BCG vaccination alone. Despite the lack of protective efficacy over BCG, a 2016 correlates analysis found that the presence of Ag85A-specific IgG titers correlated with a reduced risk of developing TB (53), pointing to the unexpected presence of a humoral immune correlate of protection in this human vaccine study.

More recently, protection was observed using an adjuvanted fusion-protein vaccine strategy in the M72/AS01_E_ vaccine trial (54). The trial tested the safety and protective efficacy of a vaccine comprised of a fusion of *Mtb* antigens *Mtb*32A and *Mtb*39A, two mycobacterial virulence factors poised to induce robust T cell immunity, delivered with AS01 as the adjuvant. This phase 2b trial showed an exciting 54% protection against progression to active tuberculosis disease in *Mtb*-infected adults. Interestingly, while expected T cell immunity was observed, the vaccine induced a robust anti-M72 protein-specific IgG response that remained 26-fold higher than the pre-vaccination levels even 12 months following vaccination (55). Given that these proteins may contribute to mycobacterial virulence, it is plausible that both canonical virulence blocking antibodies as well as potentially non-canonical antibody functions could contribute to vaccine efficacy. Thus, additional work is required to identify more precisely the potential mechanistic role of antibodies in M72-mediated protection from infection.

Overall, data from vaccine studies in mice, primates, and humans, have hinted at a potentially protective role for antibodies in mediating protection. While it must be acknowledged that in each of these cases a robust T cell response was also observed, the antibody responses should not be overlooked and may function independently of, and/or in coordination with CMI to confer protection. Thus, vaccines designed to induce antigen-specific immunoglobulins merit further consideration.

## Future Perspectives for Antibodies

**Figure 2:**
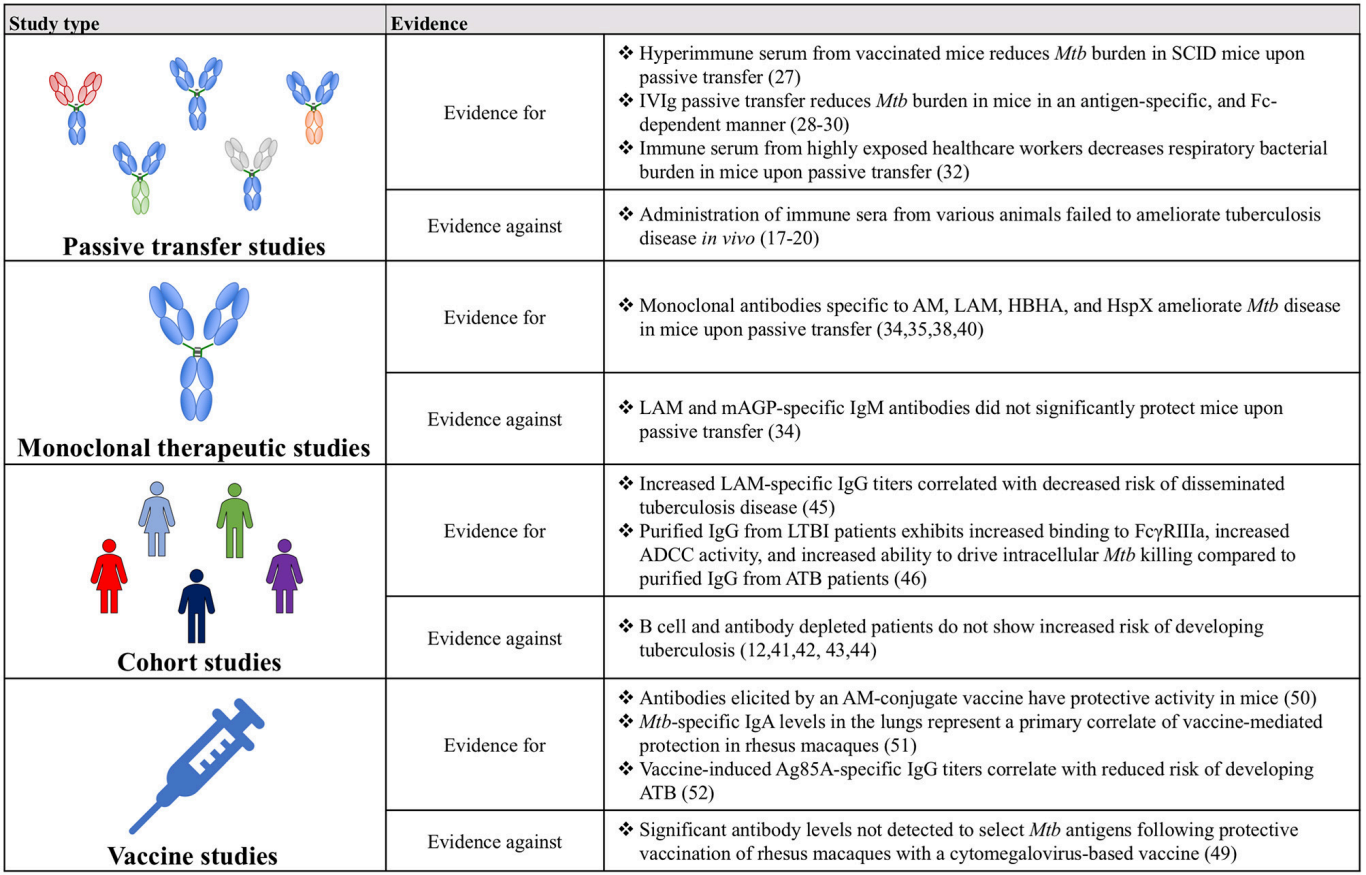
A table summarizing the key findings for and against a role for antibodies in *Mtb* disease control.

Together, the studies examined above illustrate that antibodies can modulate *Mtb* disease *in vivo*, and that this protective effect can manifest in numerous ways (Figure 2). Across numerous categories of experimental evidence, antibodies binding specific antigens, including polysaccharide surface antigens (AM and LAM), and virulence factors (HBHA and Ag85) demonstrate particular promise. Nevertheless, relative to the large number and broad landscape of *Mtb* antigens, very little is known about additional antigens that may represent productive antibody targets. Moving forward, continued generation and testing of *Mtb*-specific monoclonal antibodies against a diverse array of *Mtb* antigens will allow this further antigenic characterization and the evolution of vaccine design, beyond the empirical approach, to a rational process founded on knowledge of protective targets in *Mtb*.

Beyond recognition of specific antigens by the antibody variable domain, evidence indicates that antibody Fc-mediated signaling also plays a critical part in *Mtb* control. Mice unable to signal through activating Fc-gamma receptors have increased bacterial burden in the lung and spleen following *Mtb* infection, as well as decreased survival compared to wild-type mice (56). Consistent with this finding, lines of evidence across passive transfer, cohorts, and vaccine studies all indicate a likely role for the antibody Fc in antibody-mediated protection (40, 45–47, 56). As alluded to above, this Fc-mediated action may function through enhanced opsonize bacterial uptake, through the recruitment of innate immune cell killing of infected macrophages, or through the potentiation of adaptive immunity through the delivery of antigens for immune priming via antigen presentation (Figure 1) (51). Expanding our understanding of the mechanisms by which antibodies may selectively leverage distinct immune effector mechanisms of action will be critical for the rational design of next generation monoclonals or vaccines to combat *Mtb* disease.

Future studies leveraging animal models will remain key to uncover antibody mechanisms of action, and will advance the development of vaccines and therapeutics able to fully harness the humoral immune system to fight the bacteria. Non-human primates represent the ideal animal model for antibody studies due to similar disease manifestations (spectrum of disease) as observed in humans (57), as well as their highly homologous Fc-receptor repertoire and function (58). However, due to the limited number of BL3 facilities able to conduct *Mtb* infections in NHPs, as well as the high cost associated with running NHP studies, the model remains under-utilized. Nevertheless, mice represent an imperfect but potential first model to study the role of antibodies in *Mtb* disease. While mice do not display the same spectrum of tuberculosis infection states as humans, numerous consistencies between mouse and human work have advocated for their continued use. For example, LAM-specific antibodies have been demonstrated to ameliorate *Mtb* disease in mice (35), and in humans, high LAM antibody titers are associated with less severe tuberculosis disease (45). Moreover, Li et al. demonstrated that antibodies from latently infected, and highly exposed, but uninfected healthcare workers reduced *Mtb* burden in mice upon passive transfer as compared to IgG from ATB patients, mimicking results observed *in vitro* in an *Mtb* human whole-blood restriction assay (32). Furthermore, given the analogous response of human and innate immune cells to human IgG1 antibodies (59), the mouse model offers an opportunity to explore the mechanistic function of anti-*Mtb* IgG antibodies *in vivo*. Finally, human Fc-receptor knock-in mice, able to respond to both IgGs (60) and IgAs (40) offer additional opportunities to screen and explore human protective antibody responses *in vivo*.

## Concluding Remarks

Though historically humoral immunity has often been overlooked, and in some instances wrongfully discredited, in the context of *Mtb* infection, the evidence amassed from passive transfer, monoclonal therapeutics, cohort and vaccine studies has coalesced into a compelling argument for the importance of antibodies and for their continued study (Figure 2). Ultimately, expanding on this work will provide a more complete picture of the immunological drivers of protection against *Mtb*, beyond simple CMI, and may precipitate the development of novel therapeutics and vaccines against this global killer.

## Author Contributions

JK was the primary author of the manuscript. EI and GA offered substantial writing support, edits, and suggestions.

### Conflict of Interest Statement

The authors declare that the research was conducted in the absence of any commercial or financial relationships that could be construed as a potential conflict of interest.
